# Quenching of the Eu^3+^ Luminescence by Cu^2+^ Ions in the Nanosized Hydroxyapatite Designed for Future Bio-Detection

**DOI:** 10.3390/nano11020464

**Published:** 2021-02-11

**Authors:** Katarzyna Szyszka, Sara Targońska, Agnieszka Lewińska, Adam Watras, Rafal J. Wiglusz

**Affiliations:** 1 Institute of Low Temperature and Structure Research, PAS, Okolna 2, 50-422 Wroclaw, Poland; s.targonska@intibs.pl (S.T.); a.watras@intibs.pl (A.W.); 2 Faculty of Chemistry, University of Wroclaw, Joliot-Curie 14, 50-383 Wroclaw, Poland; agnieszka.lewinska@chem.uni.wroc.pl; 3 International Institute of Translational Medicine, Jesionowa 11 St., 55–124 Malin, Poland

**Keywords:** apatite, europium ions, cooper ions, photoluminescence spectroscopy, EPR spectroscopy

## Abstract

The hydroxyapatite nanopowders of the Eu^3+^-doped, Cu^2+^-doped, and Eu^3+^/Cu^2+^-co-doped Ca_10_(PO_4_)_6_(OH)_2_ were prepared by a microwave-assisted hydrothermal method. The structural and morphological properties of the products were investigated by X-ray powder diffraction (XRD), transmission electron microscopy techniques (TEM), and infrared spectroscopy (FT-IR). The average crystal size and the unit cell parameters were calculated by a Rietveld refinement tool. The absorption, emission excitation, emission, and luminescence decay time were recorded and studied in detail. The ^5^D_0_ → ^7^F_2_ transition is the most intense transition. The Eu^3+^ ions occupied two independent crystallographic sites in these materials exhibited in emission spectra: one Ca(1) site with C_3_ symmetry and one Ca(2) sites with C_s_ symmetry. The Eu^3+^ emission is strongly quenched by Cu^2+^ ions, and the luminescence decay time is much shorter in the case of Eu^3+^/Cu^2+^ co-doped materials than in Eu^3+^-doped materials. The luminescence quenching mechanism as well as the schematic energy level diagram showing the Eu^3+^ emission quenching mechanism using Cu^2+^ ions are proposed. The electron paramagnetic resonance (EPR) technique revealed the existence of at least two different coordination environments for copper(II) ion.

## 1. Introduction

Apatite-type materials can be applied in many industrial fields, e.g., as sorbents, biocompatible and biodegradable materials for bone and teeth reconstruction, catalysts, materials for the wastewater treatment, fertilizers, and luminescent materials [1,2]. Hydroxyapatite (Ca_10_(PO_4_)_6_(OH)_2_—abbr. HAp) is used in medicine as a bone implant material due to its biocompatibility, bioactivity, and similarity to bone mineral [3,4]. However, it is still widely investigated in order to improve its properties by obtaining appropriate grain size, morphology, mechanical strength, and solubility and by adding some dopants that are, e.g., naturally built into bone apatite, ions possessing antibacterial properties, or ions enabling bio-imaging [5,6]. Infections after grafting of bone implant material are a serious problem in surgery. The idea is that doping with antibacterial ions into the grafted biomaterial will prevent bacterial biofilm formation and infection development. Inorganic antibacterial agents possess advantages such as stability and safety. The antibacterial agents include ions such as copper, silver, and zinc [7,8,9], and several studies have shown that they can play important roles in the prevention or minimization of initial bacterial adhesion [10,11]. Metal ions can react with microbial membrane, causing structural changes and permeability. Then, Ag^+^ and Cu^2+^ ions have the ability to complex anions such as –NH_2_, –S–S–, and –CONH– of the proteins or enzymes in the bacterial cells. It provides bacterial DNA and RNA damage and inhibits proliferation [7,11,12]. Copper is an essential microelement that is involved in many metabolic processes that taken place in human bodies [7,11,13,14]. However, copper ions may have potentially toxic effects at higher amount in human beings due to their ability to generate ROSs (Reactive Oxygen Species). On the other hand, recent studies have shown that copper-doped apatite-type materials are very promising as a new kind of ow-toxic pigment that can be used in the paint and varnish industry [1,15,16,17,18].

Lanthanide(III)-doped nanomaterials are promising candidates for fluorescent bio-labels due to their stable luminescence over time, high photochemical stability, sharp emission peak, low levels of photobleaching, and toxicity compared with organic fluorophores. Eu^3+^ ions are structural and luminescence probe-sensitive to changes in the local environment around the ion. Furthermore, the luminescence of Eu^3+^ ions are identified by a narrow emission band and long lifetimes of the excited state [19,20].

Apatite is a big family of compounds, and it is widely investigated due to its outstanding properties such as good biocompatibility or possibility to be doped with different ions in a broad concentration range for applications in the industry, in medicine, etc. There are a lot of papers focusing on apatite synthesis [21,22,23,24]; its doping with antibacterial ions [9,10,25]; as well as its doping with luminescence ions such as Eu^3+^ [5,19,26,27], Tb^3+^ [28,29], Eu^3+^/Tb^3+^ [30], Er^3+^/Yb^3+^, or Eu^3+^/Cu^2+^ [17]. Moreover, there is a lot of research focusing on anion-substituted apatite such as silicate [31,32], vanadate [33], borate [34], or carbonate [35].

In the presented work, the synthesis, structural, morphological, and luminescence properties of Eu^3+^/Cu^2+^ co-doped HAp were investigated attentively. To the best of our knowledge, this is the first time that the quenching mechanism in this system has been elucidated.

## 2. Materials and Methods

### 2.1. Synthesis

The Ca_10_(PO_4_)_6_(OH)_2_ nanopowders doped with Eu^3+^ and Cu^2+^ ions were synthesized by a microwave-assisted hydrothermal method. The starting materials used were CaCO_3_ (99.0%, Alfa Aesar, Karlsruhe, Germany), NH_4_H_2_PO_4_ (99.0%, Fluka, Bucharest, Romania), Eu_2_O_3_ (99.99%, Alfa Aesar, Karlsruhe, Germany), Cu(NO_3_)_2_∙2.5H_2_O (98.0–102.0%, Alfa Aesar, Karlsruhe, Germany), and NH_3_∙H_2_O (99%, Avantor, Gliwice, Poland) as a pH regulation reagent. The concentration of dopants was calculated based on inductively coupled plasma-optical emission spectrometer (ICP-OES) results. The concentrations of europium ions were 0.5 mol%, 1 mol%, 2 mol%, and 5 mol%, and the concentrations of the cooper ions were 2 mol% and 5 mol% to the overall molar content of calcium cations. First, the stoichiometric amounts of CaCO_3_ as well as Eu_2_O_3_ were separately digested in excess of HNO_3_ (suprapur Merck, Darmstadt, Germany) to obtain water-soluble nitrates. The obtained europium nitrate hydrate was recrystallized three times to remove excess HNO_3_. Then, the stoichiometric amount of Eu(NO_3_)_3_ was dissolved in deionized water, and then, the Cu(NO_3_)_2_ was added to the stoichiometric amount of calcium nitrate. After this, NH_4_H_2_PO_4_ was added to the abovementioned mixture and the pH value was adjusted to 9 by ammonia. The suspension was transferred to a Teflon vessel and was placed into the microwave reactor (ERTEC MV 02-02, Wrocław, Poland). The reaction system was heat-treated at 280 °C for 90 min under autogenous pressure of 60 atm. The obtained product was washed several times with deionized water and dried at 70 °C for 24 h.

### 2.2. Powder Characterization

The crystal structure and phase purity were studied using a PANalytical X’Pert Pro diffractometer (Malvern Panalytical Ltd., Malvern, UK) equipped with Ni-filtered Cu Kα radiation (V = 40 kV and I = 30 mA). The recorded X-ray powder diffraction patterns (XRD) were compared with the reference standard of hexagonal calcium hydroxyapatite (P6_3_/m) from the Inorganic Crystal Structure Database (ICSD-2866) and analyzed. Rietveld structural refinement was performed with the aid of a Maud program (version 2.93) (University of Trento-Italy, Department of Industrial Engineering, Trento, Italy) [36,37] based on the apatite hexagonal crystal structure with better approximation and indexing of the Crystallographic Information File (CIF). The quality of structural refinement was checked by R-values (R_w_, R_wnb_, R_all_, R_nb_, and σ), which were followed to get a structural refinement with better quality and reliability.

The morphology was investigated by high-resolution transmission electron microscopy (HRTEM) using a Philips CM-20 SuperTwin microscope (Eindhoven, The Netherlands), operating at 200 kV. The specimen for the HRTEM measurement was obtained by dispersing a small amount of powder in methanol and by putting a droplet of the suspension onto a copper microscope grid covered with carbon.

Fourier transform infrared spectra were measured using a Thermo Scientific Nicolet iS50 FT-IR spectrometer (Waltham, MA, USA) in the range of 4000–400 cm^−1^ at 295 K. Absorption spectra were recorded with an Agilent Cary 5000 spectrophotometer, employing a spectral bandwidth (SBW) of 0.1 nm in the visible and ultraviolet range and of 0.7 nm in the infrared. The spectra were recorded at room temperature.

The excitation spectra were recorded with the aid of an FLS980 Fluorescence Spectrometer (Edinburgh Instruments, Kirkton Campus, UK) equipped with 450 W Xenon lamp. The excitation of 300 mm focal length monochromator was in Czerny–Turner configuration and the excitation arm was supplied with holographic grating of 1800 lines/mm grating blazed at 250 nm. The excitation spectra were corrected to the excitation source intensity. The emission spectra were measured by using a Hamamatsu PMA-12 photonic multichannel analyzer (Hamamatsu, Hamamatsu City, Japan) equipped with BT-CCD line (Hamamatsu, Hamamatsu City, Japan). As an excitation source, a pulsed 266 nm line of Nd:YAG laser (3rd harmonic; LOTIS TII, Minsk, Belarus) was chosen (ƒ = 10 Hz, t < 10 ns). The detection setup was calibrated and had a flat response for the whole working range (350–1100 nm). The measurements were carried out at 300 K.

The time-resolved luminescence spectrum was obtained by recording decay curves during changing observed wavelength and by creating a two-dimensional map (i.e., intensity vs. time and wavelength). It was recorded by an in-house developed software that controlled the equipment. A Dongwoo Optron DM711 monochromator (Hoean-Daero, Opo-Eup, Gyeonggi-Do, Korea) with a focal length of 750 mm was used to select the observed wavelength, while luminescence decay curves were acquired with a Hamamatsu R3896 photomultiplier (Hamamatsu, Hamamatsu City, Japan) connected to a digital Tektronix MDO 4054B oscilloscope (Bracknell, UK). An optical parametric oscillator Opotek Opolette 355 LD (Carlsbad, CA, USA) emitting 5 ns pulses was used as an excitation source.

The luminescence kinetics were measured by using a Jobin-Yvon THR1000 monochromator (HORIBA Jobin-Yvon, Palaiseu, France) equipped with a Hamamatsu R928 photomultiplier (Hamamatsu, Hamamatsu City, Japan) as a detector and a LeCroy WaveSurfer as a digital oscilloscope (Teledyne LeCroy, Chestnut Ridge, NY, USA). As an excitation source, a pulsed 266 nm line from an Nd:YAG laser was used. The luminescence kinetics were monitored at 618 nm according to the most intense electric dipole transition (^5^D_0_ → ^7^F_2_), and the effective emission lifetimes were calculated using the following equation:

(1) $$\tau_{m}=\frac{\int_{0}^{\infty }tI(t)dt}{\int_{0}^{\infty }I(t)dt}≅\frac{\int_{0}^{t_{\max}}tI(t)dt}{\int_{0}^{t_{\max}}I(t)dt}$$

where *I*(*t*) is the luminescence intensity at time *t* corrected for the background and the integrals are calculated over the range of 0 < *t* < *t*_max_, where *t*_max_ >> *τ_m_*.

The effective content of elements was determined by using an Agilent 720 bench-top optical emission spectrometer with inductively coupled Ar plasma (Ar-ICP-OES) and was corrected to an effective value. The ICP standard solutions were used to record the calibration curves to determine the Ca^2+^, P^5+^, Cu^2+^, and Eu^3+^ ion content. The samples for elemental analysis were prepared by digesting in the pure HNO_3_ acid (65% suprapur Merck).

The electron paramagnetic resonance (EPR) spectra were measured at 295 K and 77 K using a Bruker Elexsys 500 CW-EPR (Bruker GmbH, Rheinstetten, Germany) spectrometer operating at the X-band frequency (≈9.7 GHz), equipped with frequency counter (E 41 FC) and NMR teslameter (ER 036TM). The spectra were measured with a modulation frequency of 100 kHz, microwave power of 10 mW, modulation amplitude of 10 G, time constant of 40 ms, and a conversion time of 160 ms. The first derivative of the absorption power was recorded as a function of the magnetic field value. An analysis of the EPR spectra was carried out using the WinEPR software package, version 1.26b (Bruker WinEPR GmbH, Rheinstetten, Germany).

## 3. Results and Discussion

### 3.1. Structural Analysis

**Figure 1:**
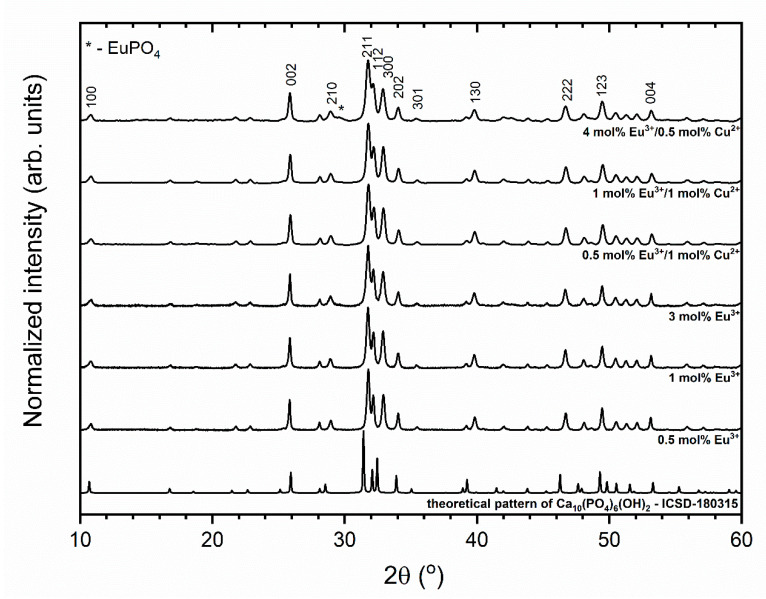
X-ray powder diffraction patterns of the Eu^3+^-doped and Eu^3+^/Cu^2+^ co-doped Ca_10_(PO_4_)_6_(OH)_2_.

The structural characterization of the HAp nanocrystals doped with xEu^3+^ (where x = 0.5, 1, and 3 mol%) and co-doped with xEu^3+^ and yCu^2+^ (where x = 0.5, 1, and 4 mol% and y = 0.5 and 1 mol%) was carried out by powder X-Ray diffraction measurements as a function of doping ion concentration (see Figure 1). Detectable crystallinity and pure hexagonal phase corresponding to the reference standard (ICSD—180315 [38]) were observed. Only in the case of the 4 mol% Eu^3+^/0.5 mol% Cu^2+^:HAp, an extra peak at 29.5° of 2θ was observed (assigned as asterisk in Figure 1).

**Figure 2:**
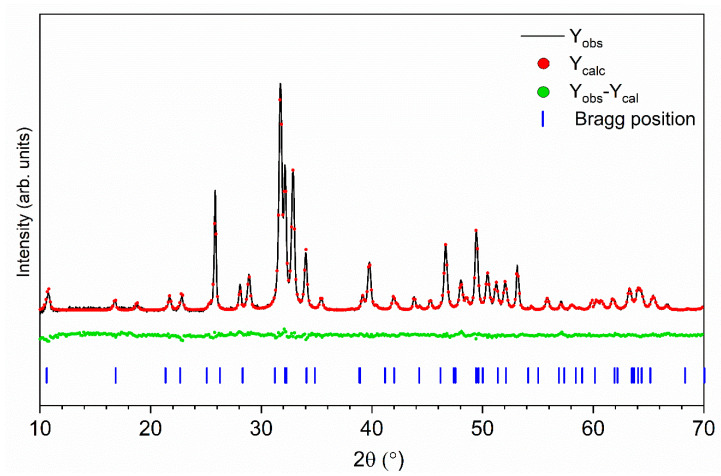
Representative results for the 0.5 mol% Eu^3+^/1 mol% Cu^2+^:Ca_10_(PO_4_)_6_(OH)_2_:, Rietveld analysis (red—fitted diffraction, green—differential pattern, and blue column—reference phase peak position).

Structural refinement was performed to obtain the unit cell parameters and the average grain sizes of synthesized materials. Hexagonal phase formation and the successful incorporation of Eu^3+^ and Cu^2+^ ions were verified. The theoretical fit with the observed XRD pattern was found to be in good agreement, which indicated the success of the Rietveld refinement method (see Figure 2). More details are displayed in Table 1. As can be seen, it was possible to observe an increase in the cell volume and a parameters with the increase in Eu^3+^ ion concentration in single-doped materials, which was caused by a smaller ionic radii of the dopant (Ca^2+^ (coordination number—CN9), 1.18 Å; Eu^3+^ (CN9), 1.12 Å; Ca^2+^ (CN7), 1.06 Å; and Eu^3+^ (CN7), 1.01 Å) [39]. Moreover, shrinkage of the average grain size with an increase in the Eu^3+^ ion concentration in the host lattice was observed. In the case of co-doped materials, no straightforward dopant concentration dependence on cell parameters (a, c, and V) or average grain size was observed.

**Figure 3:**
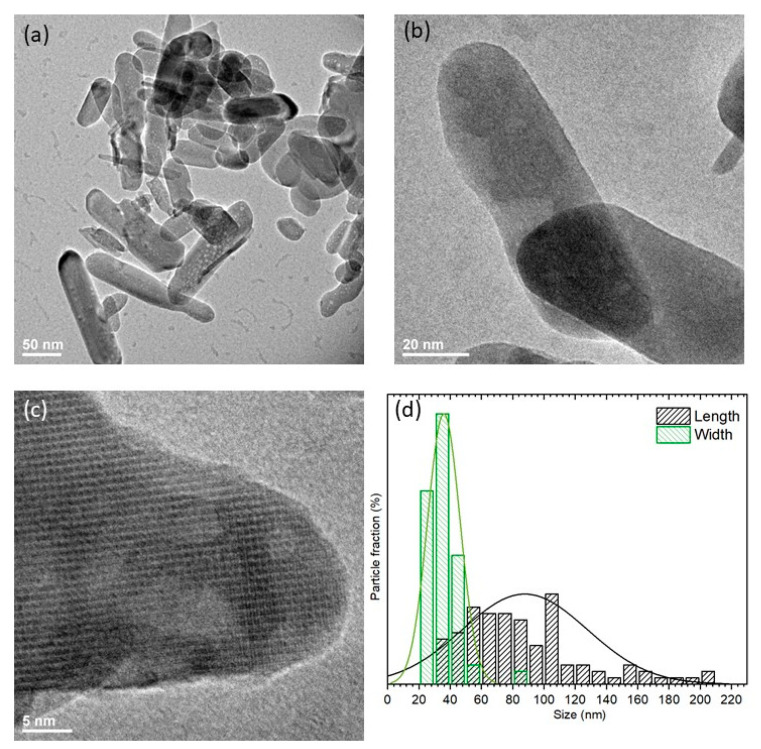
Representative TEM images (**a**–**c**) and particle size distribution (**d**) of the 1 mol% Eu^3+^:Ca_10_(PO_4_)_6_(OH)_2_.

The morphology of the calcium hydroxyapatite was investigated by HRTEM. Nanoparticles are crystalline in nature and elongated, as can be seen in Figure 3. The particle size distribution is relatively wide, and the mean grain sizes of particle is in the range between 60 and 120 nm in length and about 40 nm in width.

**Figure 4:**
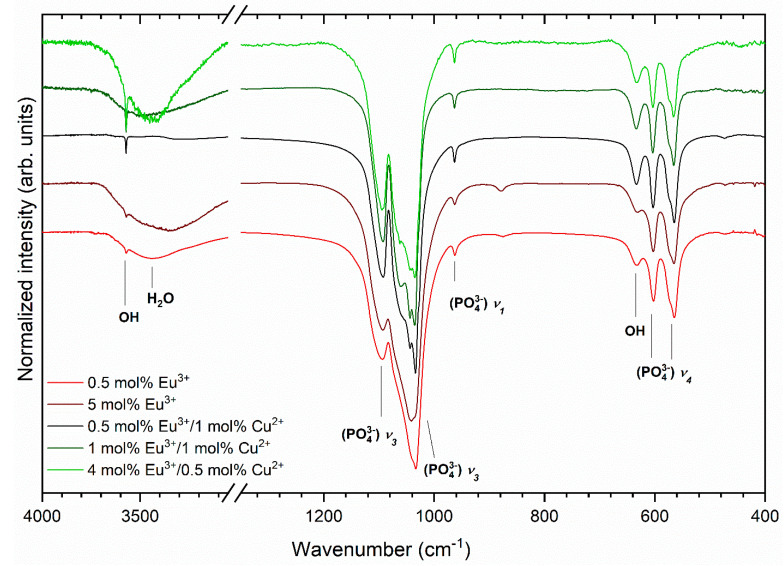
Infrared spectra of the Eu^3+^-doped and Eu^3+^/Cu^2+^ co-doped Ca_10_(PO_4_)_6_(OH)_2_.

The infrared spectra of the copper-doped, europium-doped, and co-doped hydroxyapatite materials are presented in Figure 4. The most intense peaks are the triply degenerated antisymmetric stretching bands of phosphate groups ν_3_(PO_4_^3−^) located at 1044.5 cm^−1^ and 1097.8 cm^−1^. The peaks observed at 566.0 cm^−1^ and 603.1 cm^−1^ correspond to the triply degenerated ν_4_(PO_4_^3−^) vibrations. The peaks at 963.0 cm^−1^ are assigned to the non-degenerated symmetric stretching ν_1_(PO_4_^3−^) band. Two peaks corresponding to OH^−^ group at 3571.5 cm^−1^ and 633.5 cm^−1^ are observed on the infrared spectra. The existence of these peaks clearly confirms the hydroxyapatite structure with a hydroxyl group in the host lattice. The broad bands between 3690 and 3290 cm^−1^ were connected with H_2_O vibration.

### 3.2. Absorption, Excitation, and Emission Spectra

**Figure 5:**
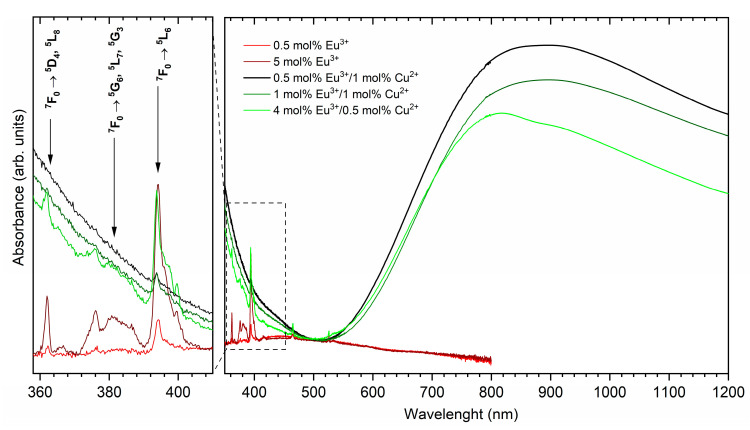
The absorption spectra of the Eu^3+^-doped and Eu^3+^/Cu^2+^-co-doped Ca_10_(PO_4_)_6_(OH)_2_.

The absorption spectra of the pure, copper-doped, europium-doped, and co-doped hydroxyapatite nanopowders were recorded in the visible range from 350 nm to 800 nm at room temperature (see Figure 5). The pure hydroxyapatite matrix is transparent for these wavelengths. The copper-doped materials absorbed the blue radiation in the range from 350 nm to 420 nm. All materials are relatively transparent for the radiation from 450 nm to 550 nm of the wavelength. The copper-doped materials absorbed the radiation from 550 nm to 800 nm, and the absorption coefficient increases with the increase in wavelength. The broad absorption band is attributed to the ^2^E → ^2^T_2_ intra-configurational (d-d) transition of the Cu^2+^ ions [40,41]. In the absorption spectra, the peaks related to the 4f-4f transitions of Eu^3+^ ions are observed. These peaks are attributed to the following transitions: the ^7^F_0_ → ^5^D_4_, ^5^L_8_ at 362 nm and ^7^F_0_ → ^5^G_6_, ^5^L_7_, ^5^G_3_ at 376 nm. The ^7^F_0_ → ^5^L_6_ transition with a maximum at 394 nm was observed in the case of europium and the copper co-doped materials. This transition is the most intense f-f transition of Eu^3+^ ions.

**Figure 6:**
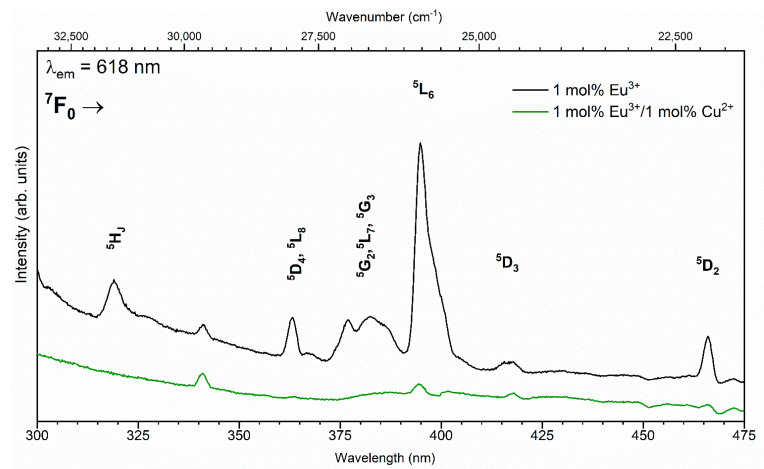
The excitation spectra of the 1 mol% Eu^3+^ and 1 mol% Eu^3+^/1 mol% Cu^2+^-co-doped Ca_10_(PO_4_)_6_(OH)_2_.

The excitation emission spectra, which were recorded at room temperature by monitoring the intense red emission at 618 nm (^5^D_0_ → ^7^F_2_), of investigated materials are presented in Figure 6. The representative excitation spectra of the 1 mol% Eu^3+^:HAp and 1 mol% Eu^3+^/1 mol% Cu^2+^:HAp are presented. As demonstrated, the excitation spectra consisted of visible intra-configurational 4f-4f transitions with sharp lines characteristic of Eu^3+^ ions. Particularly, these narrow bands located at around 320, 363, 383, 395, 416, and 466 nm originated from the ^7^F_0_ → ^5^H_J_; ^7^F_0_ → ^5^D_4_, ^5^L_8_; ^7^F_0_ → G_2_, ^5^L_7_, ^5^G_3_; ^7^F_0_ → ^5^L_6_; ^7^F_0_ → ^5^D_3_ transitions of Eu^3+^ ions, respectively [31,35]. The absorption peak of the ^7^F_0_ → ^5^H_J_ transition at 320 nm indicates that the energy band-gap of HAp is considerably larger than that in, e.g., Eu_2_Ti_2_O_7_ oxide [42], in which the ^5^H_3,6_-related transition peak is completely masked by the charge transfer band due to its lower energy band-gap nature. The f-f electron transitions are weakly affected by the crystal field; thus, their positions remain almost steady due to good isolations of lanthanide’s f orbitals by an external shell [19,31,43]. As can be seen, the intensity of the emission excitation spectra is much lower in the case of the co-doped material than in that single doped with Eu^3+^ in the HAp host.

The spectroscopic properties of Eu^3+^ ions allow us to receive vital information about the symmetry of the Eu^3+^ ions surrounding the crystal lattice; the amount of crystallographic positions; and therefore, potential sites of substitution, structural changes occurring in the matrix caused by external factors, etc. The emission spectra of the Eu^3+^ ions consist of characteristic bands present in the red region of the electromagnetic radiation assigned to the electron transitions developing in the 4f-4f shell of Eu^3+^ ions. The ^5^D_0_ → ^7^F_0,1,2_ transitions are the most important in analysis, particularly in correlation with the structural properties. The ^5^D_0_ → ^7^F_0_ transition can provide direct information about the number of crystallographic sites occupied by Eu^3+^ ions in the host lattice [19].

**Figure 7:**
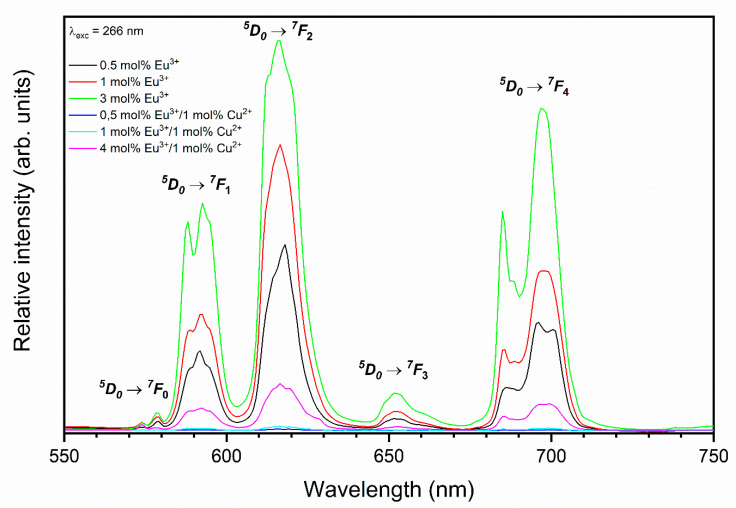
The emission spectra of the Eu^3+^-doped and Eu^3+^/Cu^2+^ co-doped Ca_10_(PO_4_)_6_(OH)_2_.

The emission spectra of y mol% Eu^3+^:HAp (where y—0.5, 1, and 3 mol%) and x mol% Eu^3+^/y mol% Cu^2+^:HAp (where x—0.5, 1, and 4 mol% and y—0.5 and 1 mol%) were measured by excitation wavelength at 266 nm at room temperature and are shown in Figure 7. The spectra were normalized to the ^5^D_0_ → ^7^F_1_ magnetic dipole transition. The emission spectra were dominated by an intense red emission band situated at about 618 nm corresponding to the ^5^D_0_ → ^7^F_2_ transition of Eu^3+^ ions. Meanwhile, four weaker emission bands peaking at around 578, 589, 652, and 698 nm were also detected and ascribed to the ^5^D_0_ → ^7^F_0_, ^5^D_0_ → ^7^F_1_, ^5^D_0_ → ^7^F_3_, and ^5^D_0_ → ^7^F_4_ transitions of Eu^3+^ ions, respectively [31,35]. The presence of a ^5^D_0_ → ^7^F_0_ transition confirms that europium ions are located in a low-symmetry environment. Furthermore, the number of lines directly indicate the number of occupied crystallographic positions in the investigated lattice by Eu^3+^ ions. In the apatite molecule, ten calcium atoms are found in two non-equal crystallographic positions, in agreement with the results showed in Figure 7.

**Figure 8:**
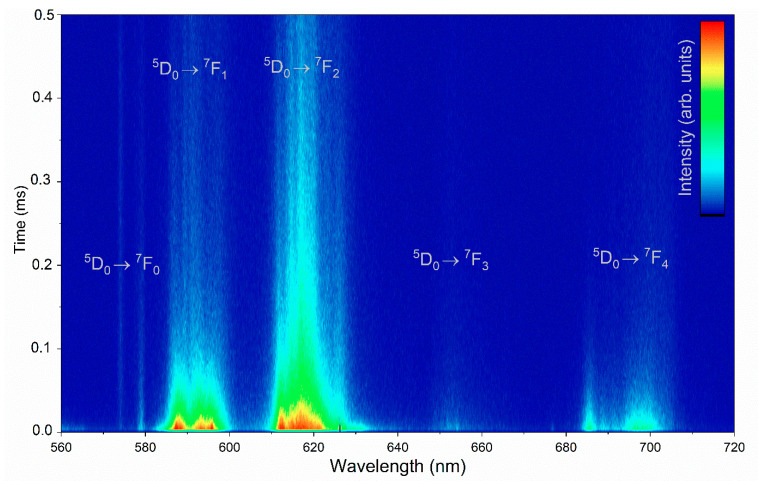
Representative emission spectra map of the 1 mol% Eu^3+^/1 mol% Cu^2+^:Ca_10_(PO_4_)_6_(OH)_2_.

In Figure 8, the time-resolved emission spectrum of the 1 mol% Eu^3+^/1 mol% Cu^2+^:HAp is presented.

### 3.3. Decay Profiles

**Figure 9:**
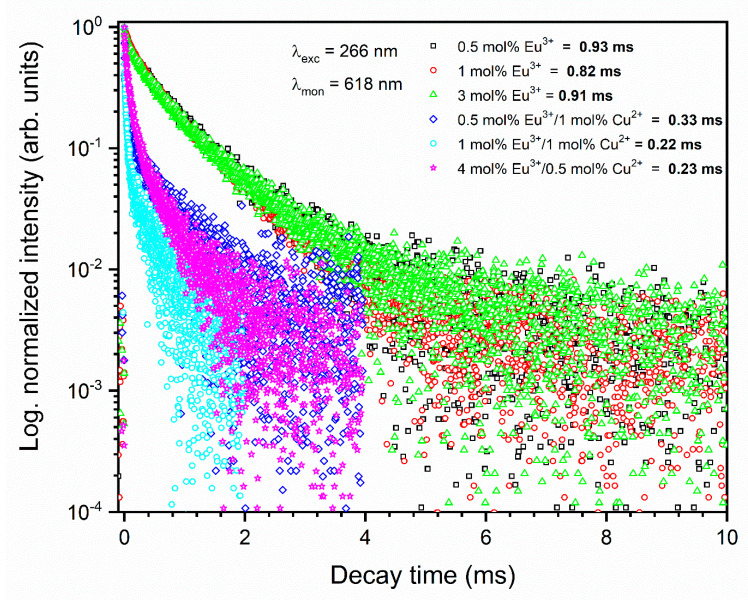
Decay times of the Eu^3+^-doped and Eu^3+^/Cu^2+^ co-doped Ca_10_(PO_4_)_6_(OH)_2_.

The luminescence decay curves were registered and analyzed for the synthesized materials to determine the comprehensive characteristics of the luminescence properties. The decay curves presented in Figure 9 are not single-exponential, which is compatible with the presence of nonequivalent crystallographic sites of Eu^3+^ ions accordingly. The lifetimes values were calculated as the effective emission decay time by using Equation (1). The average lifetimes obtained for the sample single-doped by Eu^3+^ are equal to 0.93; 0.82, and 0.91 for concentrations of optically active ions at 0.5, 1.0, and 3.0 mol%, respectively.

The emission kinetic of Eu^3+^ ions strongly depends on the presence of Cu^2+^ ions, which effectively quenched the ^5^D_0_ level. The average lifetime obtained for co-doped materials is much shorter than that for Eu^3+^-doped materials, and the decay time values are estimated at about 0.33, 0.22, and 0.23 ms for the 0.5 mol% Eu^3+^/1 mol% Cu^2+^, 1 mol% Eu^3+^/1 mol% Cu^2+^, and 4 mol% Eu^3+^/0.5 mol% Cu^2+^ co-doped materials, respectively. The emission quenching of the Eu^3+^ ions may be interpreted as nonradiative energy transfer between the Eu^3+^ and Cu^2+^ ions. The efficiency of energy transfer was estimated by Equation (2) for pairs of materials: single-doped with Eu^3+^ ions and co-doped with Eu^3+^ and Cu^2+^ ions with the same concentration of Eu^3+^ ions. The calculated lifetimes of Eu^3+^ ions (donor) in the absence and presence of Cu^2+^ ions (acceptor) are used [40,44]. The results of energy transfer efficiency are presented in Table 2.

(2) $$\eta_{{\mathrm{Eu}}^{3+}\to {\mathrm{Cu}}^{2+}}=1-\left(\frac{\tau_{{\mathrm{Eu}}^{3+}\to {\mathrm{Cu}}^{2+}}}{\tau_{{\mathrm{Eu}}^{3+}}}\right)$$

The obtained efficiency is equal to 65 and 73% for 0.5 mol% Eu^2+^/1 mol% Cu^2+^ and 1 mol% Eu^2+^/1 mol% Cu^3+^ co-doped HAp, respectively. The observed luminescence properties of europium ions in apatite lattice in the presence of copper ions are dominated by emission quenching of Eu^3+^ by Cu^2+^ ions. With an increase in Eu^3+^ concentration, the efficiency of quenching grew. This would suggest that the relatively huge probability of Eu^3+^ → Cu^2+^ nonradiative energy transfer probably behaves by electric dipole interaction [40,44].

**Figure 10:**
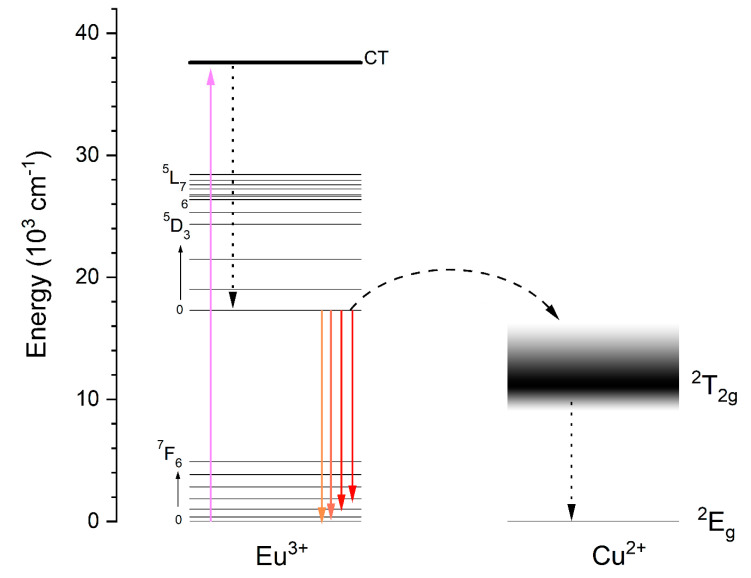
Simplified energy level scheme of Eu^3+^ and Cu^2+^ explaining quenching of Eu^3+^ ion emission.

The simplified energy level diagram of Eu^3+^ and Cu^2+^ ions was proposed and is shown in Figure 10 in order to explain the quenching mechanism occurring in hydroxyapatite co-doped with Eu^3+^ and Cu^2+^ ions. When the materials were excited by 266 nm wavelength, a charge transfer transition O^2−^ → Eu^3+^ occurred. Then, nonradiative relaxation to the ^5^D_0_ first excited state was performed. From this state, the energy can be relaxed in two ways: by radiative transition to the ground state of Eu^3+^ ions (^7^F_0–6_) or by energy transfer to the ^2^T_2g_ energy level of Cu^2+^ and then nonradiative relaxation to the ground state of Cu^2+^ ions. These two manners of energy relaxation compete among themselves, and doping with Cu^2+^ ions causes Eu^3+^ emission quenching.

### 3.4. The EPR Spectra Analysis

The EPR spectroscopy is especially predisposed to identifying the structural properties of paramagnetic compounds. The unpaired electron interacts (couples) with the nuclear spin (I) to form a 2I + 1 line hyperfine structure centered on g and spaced with the distance quantified by the hyperfine coupling parameter A. The coupling between the nuclear and electron spins becomes stronger as the A parameter becomes larger. The combination of g and A parameters can be utilized to differentiate between electron environments of ion.

There are two distinct Ca coordination sites in the HAp unit cell, that is the Ca(1) site with the Ca^2+^ ion surrounded by 9 oxygen atoms from 6 PO_4_^3−^ groups and the Ca(2) site with the Ca^2+^ ion surrounded by 7 oxygen atoms from the 5 PO_4_^3−^ and 1 OH^−^ anions. The Ca^2+^ ions in both coordination sites can be replaced by Eu^3+^ and Cu^2+^ ions [45]. The EPR properties of trivalent europium (Eu^3+^) is relatively little because it is a non-Kramer ion, and its EPR spectrum should be silent because of the short spin-lattice relaxation time [46]. Therefore, in the EPR spectra recorded for the samples, only signals due to Cu^2+^ ions are observed.

**Figure 11:**
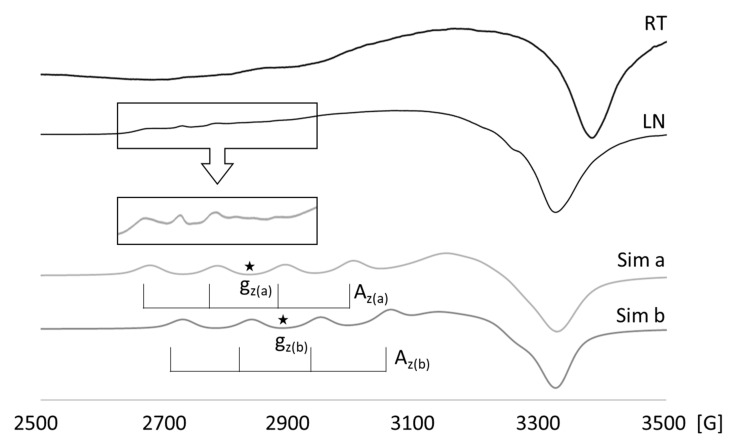
Experimental and simulated electron paramagnetic resonance (EPR) spectra of the 1 mol% Eu^3+^/1 mol% Cu^2+^:Ca_10_(PO_4_)_6_(OH)_2_.

The spectra recorded at room temperature and at 77 K (Figure 11) are anisotropic as a consequence of the Jahn–Teller effect operating for the d^9^ electron configuration of Cu^2+^ ions that leads to considerable departure from a regular symmetry of the coordination sphere. The spectra reveal a weakly resolved hyperfine interaction between the spins of unpaired electrons and copper nuclei (I = 3/2), which for powder spectrum suggest a large distance between paramagnetic centers (Cu^2+^ ions).

Spectral analysis revealed the existence of at least two different coordination environments for copper(II) ions. The EPR spectrum of the 1 mol% Eu^3+^/1 mol% Cu^2+^ co-doped HAp can be decomposed into two superimposed resonance signals due to two different Cu(II) coordination sites, hereinafter referred as “a” and “b”. This stays in line with the fact that there are two distinct Ca coordination sites in HAp in which Cu(II) can be doped. From the simulation of the spectrum recorded at 77 K, the estimated parameters are g_z(a)_ = 2.41, g_z(b)_ = 2.37, g_y_ = 2.11, and g_x_ = 2.08 with A_z(a)_ = A_z(b)_ = 110 G. However, the accuracy of these parameters is inevitably limited due to the fact that the Cu(II) signals are superimposed.

The observed EPR parameters contrast their counterparts determined for synthetic hydroxyapatite doped with Cu^2+^, in which also two different Cu(II) coordination sites were identified: g_z_ = 2.485, g_y_ = 2.17, g_x_ = 2.08, and A_z_ = 52 G for one coordination site and g_z(a)_ = 2.420, g_y_ = 2.17, g_x_ = 2.08, and A_z_ = 92 G for the second [47]. This difference in g_z_ and A_z_ parameters clearly stems from the structural divergence between the 1 mol% Eu^3+^/1 mol% Cu^2+^:HAp and SHA. At the same time, the g_z_ and A_z_ parameters for 1 mol% Eu^3+^/1 mol% Cu^2+^:HAp are similar to the ones reported for copper(II) ions bonded to lattice oxygens in montmorillonite((Cu(AlO)_n_(H_2_O)_4-n_)_x_): g_z_ and A_z_ in the ranges 2.37–2.41 and 100–140 G, respectively [48]. Therefore, the geometry of Cu(II) coordination sites in 1 mol% Eu^3+^/1 mol% Cu^2+^:HAp are expected to structurally resemble ((Cu(AlO)_n_(H_2_O)_4-n_)_x_).

Trends were found that enabled the Cu(II) EPR parameters to be correlated to the copper(II) ligands and the overall charge of the complexes [49,50,51,52]. According to these general trends, the g_z_ and A_z_ parameters for 1 mol% Eu^3+^/1 mol% Cu^2+^:HAp are characteristic of positively charged Cu–O complexes [49,50]. This fact indicates that, in the crystal lattice of the 1 mol% Eu^3+^/1 mol% Cu^2+^:HAp, the negative charge of the PO_4_^3−^ and OH^−^ anions are primarily neutralized by remained Ca^2+^ cations. Moreover, the observed difference between g_z_ values found for two Cu coordination sites can be used to determine their possible assignment to Ca(1) and Ca(2). The increase in g_z_ is associated with the rise in positive charges for the Cu–O complex. Hence, the higher value of g_z(a)_ indicates that this Cu(II) ion is surrounded by a lower number of oxygen atoms, which are the primary carriers of a negative charge, and therefore should be labeled as Cu(II) ion doped into the Ca(2) site.

## 4. Conclusions

The pure crystal hydroxyapatite powder doped co-doped with Eu^3+^ and Cu^2+^ ions was successfully synthesized by a microwave-assisted hydrothermal method that was confirmed by the X-ray powder diffraction method. The nanometric size of the obtained materials was confirmed by Rietveld refinement and TEM techniques. In the absorption spectra, the transitions occurring in Eu^3+^ as well as Cu^2+^ ions were observed. In the emission spectra, the typical transition of Eu^3+^ ions (^5^D_0_ → ^7^F_J_) were observed and the ^5^D_0_ → ^7^F_2_ transition is the most intense. The ^5^D_0_ → ^7^F_0_ transition consists of two lines, which means that the Eu^3+^ ions are localized in two independent crystallographic sites: in Ca(1) with C_3_ point symmetry and in Ca(2) with C_s_ symmetry. The emission decay times of Eu^3+^/Cu^2+^:HAp are much shorter than the decay times of Eu^3+^:HAp, which indicates that the Eu^3+^ emission is quenched by the Cu^2+^ ions. The simplified energy level diagram was proposed, and the quenching mechanism was explained. Based on the EPR measurement, the existence of at least two different coordinations surrounding copper(II) ions was detected.

## Figures and Tables

**Table 1:** Unit cell parameters (a and c), cell volume (V), grain size, as well as refine factor (R_W_) for the Eu^3+^-doped and Eu^3+^/Cu^2+^ co-doped Ca_10_(PO_4_)_6_(OH)_2_.

Sample	*a* (Å)	*c* (Å)	*V* (Å^3^)	Size (nm)	R_w_ (%)
single crystal	9.424(4)	6.879(4)	529.09(44)	–	–
doped with *x* mol% Eu^3+^					
0.5 mol% Eu^3+^	9.4139(6)	6.8905(0)	528.83(54)	55.6(2)	3.0
1 mol% Eu^3+^	9.4259(6)	6.8881(3)	530.19(10)	45.5(9)	2.8
3 mol% Eu^3+^	9.4276(8)	6.8891(5)	530.26(80)	38.0(1)	3.3
co-doped with *x* mol% Eu^3+^ and *y* mol% Cu^2+^					
0.5 mol% Eu^3+^/1 mol% Cu^2+^	9.4300(2)	6.8875(1)	530.41(48)	42.0(2)	3.1
1 mol% Eu^3+^/1 mol% Cu^2+^	9.4264(5)	6.8858(9)	529.87(91)	41.2(4)	2.7
4 mol% Eu^3+^/0.5 mol% Cu^2+^	9.4288(2)	6.8893(5)	530.41(84)	58.2(0)	3.2

**Table 2:** The average lifetime of Eu^3+^-doped (τ_Eu_), Eu^3+^/1 mol% Cu^2+^ (τ_Eu__→Cu_) co-doped Ca_10_(PO_4_)_6_(OH)_2_ and energy transfer efficiency (η_Eu__→Cu_).

	τ_Eu_ (ms)	τ_Eu_ → _Cu_ (ms)	η_Eu_ → _Cu_ (%)
0.5 mol% Eu^3+^	0.93	0.33	65
1 mol% Eu^3+^	0.82	0.22	73

## Data Availability

Not applicable.
